# A Novel Technique for Reconstruction of the Canthal Ligaments of the Lower Eyelid Using Barbed Sutures

**DOI:** 10.3390/jcm15093510

**Published:** 2026-05-04

**Authors:** Yutaro Araki, Kazuki Shimada, Miki Fujii, Takako Komiya, Hajime Matsumura

**Affiliations:** Department of Plastic and Reconstructive Surgery, Tokyo Medical University Hospital, Tokyo 160-0023, Japan; yuuutaro@tokyo-med.ac.jp (Y.A.); shimada7@tokyo-med.ac.jp (K.S.); mikidtma@gmail.com (M.F.); tkomiya@tokyo-med.ac.jp (T.K.)

**Keywords:** lower eyelid reconstruction, canthal ligament, barbed suture, ectropion, entropion, MRD-2, eyelid malposition

## Abstract

**Background/Objectives**: Lower eyelid malposition is a recognized complication following eyelid tumor excision, trauma, or degenerative changes, and is frequently associated with laxity or disruption of the canthal ligaments. Conventional reconstruction techniques using autologous grafts such as fascia lata or auricular cartilage are effective but are associated with donor-site morbidity and increased surgical complexity. This study aimed to evaluate the feasibility and early outcomes of a novel technique for reconstruction and reinforcement of the lower eyelid canthal ligaments using a barbed suture system. **Methods**: A single-institution retrospective case series was conducted, including consecutive patients who required lower eyelid canthal ligament reconstruction or horizontal support reinforcement from April 2025 to November 2025. Margin reflex distance 2 (MRD-2) was measured from standardized photographs preoperatively and at final follow-up. Munk scale scores, surgically induced astigmatism (SIA), and postoperative complications were recorded. **Results**: Seven patients (median age 72 years; range 38–86) underwent the procedure. Indications included post-oncological eyelid reconstruction (*n* = 2), cicatricial ectropion (*n* = 2), paralytic ectropion (*n* = 1), involutional ectropion (*n* = 1), and cicatricial entropion (*n* = 1). The median follow-up was 189 days (range 105–280). MRD-2 at final follow-up was 5.4 mm in Case 1 (preoperative: 5.7 mm) and 4.1 mm in Case 2 (preoperative: 4.2 mm), indicating maintained eyelid position. Munk scale scores improved in four of five evaluated patients. No recurrence of ectropion or entropion was observed during follow-up. Transient linear skin indentation along the suture pathway was observed in all cases and resolved spontaneously in all patients by 3 months postoperatively. One patient experienced transient postoperative diplopia that resolved with conservative management. **Conclusions**: This study demonstrates the feasibility of lower eyelid canthal ligament reconstruction using a barbed suture system in a heterogeneous cohort of seven patients. Short-term results are encouraging, with maintained eyelid position and no recurrence of malposition observed during the follow-up period. These preliminary findings warrant further evaluation in larger, prospective, controlled studies with longer follow-up.

## 1. Introduction

The lower eyelid is a functionally and aesthetically critical structure, and its malposition—whether ectropion, entropion, or descent—can lead to ocular surface exposure, epiphora, and significant cosmetic disfigurement [1]. The canthal ligaments, comprising the lateral and medial canthal tendons, provide essential horizontal tension that maintains the lower eyelid in its appropriate position against the globe. Dysfunction or laxity of these ligaments, whether caused by trauma, tumor resection, aging, or facial nerve palsy, is a major contributor to lower eyelid malposition [2,3].

Lower eyelid malposition is particularly common following reconstruction after excision of periocular malignancies. The anterior and posterior lamellar defects created by tumor excision, combined with disruption of canthal support, create a complex three-dimensional reconstructive challenge [3]. Standard approaches address each component separately: anterior lamellar deficits are repaired with local flaps or skin grafts, posterior lamellar deficits with hard palate mucosa or other substitute materials, and canthal laxity with horizontal tightening procedures [1].

For horizontal support reconstruction, several techniques have been described. The lateral tarsal strip procedure and its variants remain among the most widely used methods for correcting lower eyelid laxity; however, their effectiveness depends on residual tarsal integrity and may be insufficient when both lateral and medial canthal tendons are compromised [4,5]. Autologous grafts such as fascia lata or auricular cartilage have been employed for canthal tendon reconstruction, offering durable support; however, these approaches require a separate donor site, increasing operative time and morbidity [6,7]. Periosteal flap techniques offer an alternative without donor-site sacrifice, but may be limited by tissue availability and anatomical constraints [8].

STRATAFIX^®^ Spiral PDS Plus^®^ (Ethicon, LLC, Guaynabo, PR, USA) is a barbed suture system in which microscopic barbs distributed along the suture shaft provide self-anchoring properties, enabling continuous tension distribution along the suture line without reliance on knots [9]. The Spiral PDS Plus^®^ configuration features helically arranged barbs distributed circumferentially around the suture, allowing stable tissue engagement independent of suture orientation. Compared with conventional sutures, barbed sutures have been shown to cause less tissue damage at the microscopic level, making them potentially well suited for use in delicate periocular tissues [10].

To our knowledge, the use of a barbed suture system for reconstruction of the lower eyelid canthal ligaments has not been previously described, although barbed sutures have been used as temporary external fixation sutures following periocular tumor excision [11]. In this study, we present a novel technique for canthal ligament reconstruction and reinforcement and report its preliminary clinical outcomes.

## 2. Materials and Methods

### 2.1. Patients

This study was designed as a single-institution retrospective case series to describe a novel surgical technique and report its preliminary clinical outcomes and was conducted in accordance with the Declaration of Helsinki. This study was reported in accordance with the STROBE guidelines for observational studies.

Patients who visited the Department of Plastic and Reconstructive Surgery at Tokyo Medical University Hospital from April 2025 to November 2025 were considered for inclusion if reconstruction or reinforcement of the lower eyelid canthal ligaments was deemed necessary based on clinical examination. Inclusion criteria were: (1) clinically evident lower eyelid malposition (ectropion, entropion, or lower eyelid descent) or high risk of postoperative lower eyelid malposition following tumor excision; and (2) laxity or disruption of the canthal ligaments as the primary contributing factor, as assessed by the operating surgeon. Exclusion criteria were: (1) follow-up of less than 3 months; and (2) withdrawal of consent. Written informed consent for surgery and study participation was obtained from all patients.

Clinical outcomes were evaluated using electronic medical records and standardized pre- and postoperative photographs.

### 2.2. Surgical Technique

**Figure 1:**
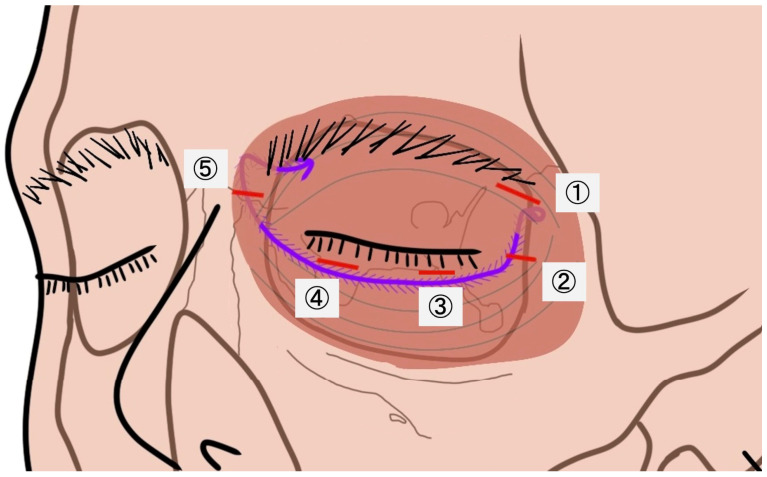
Schematic frontal illustration of the suture pathway. Red dashed lines indicate the incision sites. The purple lines and arrows indicate the barbed suture. Circled numbers indicate the five stab incisions (①–⑤) created from lateral to medial.

Surgery was performed under general or local anesthesia. Five stab incisions were created along the lower eyelid from lateral to medial and labeled ①–⑤ (Figure 1).

A 4-0 STRATAFIX^®^ Spiral PDS Plus^®^ suture was used. The factory-equipped welded loop anchor of the suture was first secured to the periosteum of the inner aspect of the lateral orbital rim through incision ①, at a point slightly superior to the lateral canthal tendon insertion corresponding approximately to the region of Whitnall’s tubercle. The anchor loop was passed through the periosteum with a single bite, and fixation adequacy was confirmed by applying gentle traction on the suture to verify secure engagement before proceeding. No additional periosteal sutures were placed. The needle was then advanced through the preseptal space beneath the orbicularis oculi muscle toward incision ②. From incision ②, the suture was passed within the sub-orbicularis plane between the orbicularis muscle and skin toward incision ③.

**Figure 2:**
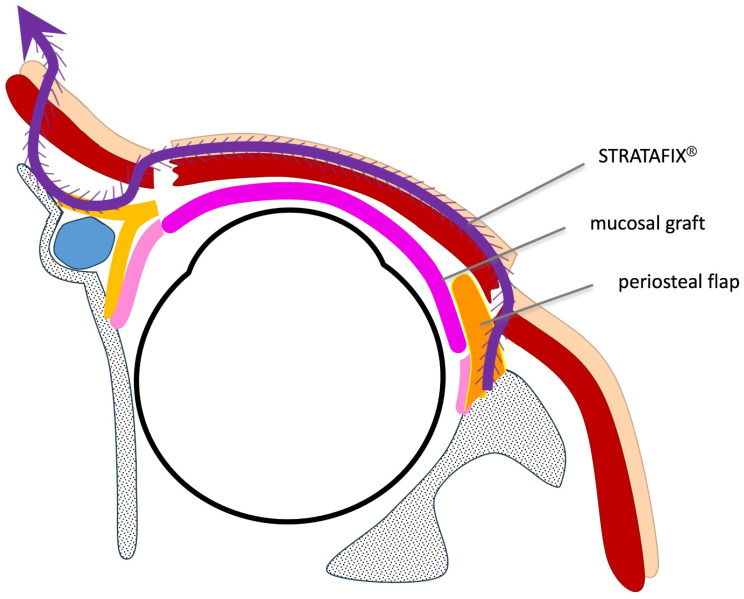
Schematic sagittal cross-section illustrating the barbed suture pathway (Case 1). The suture (purple) passes from the lateral orbital periosteum through the preseptal and suborbicularis planes to the medial periosteum, creating a continuous horizontal support vector across the lower eyelid.

From incision ③, the needle was again passed deep to the orbicularis muscle and directed to the plane anterior to the tarsal plate, corresponding to the anatomical position of the anterior limb of the medial canthal ligament, and externalized at incision ④. The periosteum of the nasofrontal bone at a point slightly superior to the medial canthal ligament insertion was then engaged, and the suture was brought out through the nasal root skin at incision ⑤. In cases of lower eyelid reconstruction following tumor excision, it is important to pass the suture through both the residual native skin and the reconstructed anterior lamella, thereby connecting the two components and ensuring continuity of the anterior lamellar support along the entire eyelid (Figure 2).

After externalization, appropriate horizontal support and apposition of the lower eyelid margin to the globe were confirmed by the operating surgeon under direct visualization. Tension was adjusted at this stage by varying the depth and extent of the final externalization pass before cutting the suture. Once satisfactory eyelid position was achieved, the suture was cut so that the end remained below the skin surface to complete the procedure. Stab incisions were closed with a single absorbable suture if necessary.

In cases in which concomitant procedures were performed, operative time was recorded as the duration of the barbed suture procedure only, from periosteal anchor fixation to suture cut, excluding the time required for concomitant procedures. In cases in which the barbed suture procedure was performed as a standalone technique, operative time was defined as the total duration from skin incision to wound closure.

### 2.3. Outcome Evaluation

The primary outcome measures differed according to the underlying indication. In post-oncological reconstruction cases, where the primary concern was maintenance of eyelid position following extensive tissue rearrangement, MRD-2 was used as the primary outcome. In patients with ectropion or entropion, where epiphora was the primary symptom, the Munk scale was used as the primary outcome. This approach reflects the different clinical goals across the heterogeneous cohort. MRD-2 was defined as the distance from the corneal light reflex to the lower eyelid margin in the primary position of gaze. MRD-2 was measured on standardized frontal photographs using ImageJ software (version 1.54m, National Institutes of Health, Bethesda, MD, USA), with pixel-to-millimeter calibration performed using the horizontal corneal diameter as a reference standard (set at 11.5 mm). All measurements were performed using anonymized images presented in random order, without knowledge of the corresponding timepoint or clinical status at the time of measurement. Measurements were performed twice, and intra-observer reliability was excellent (intraclass correlation coefficient 0.97).

In patients with ectropion or entropion, the primary outcome was the Munk scale score. The Munk scale is a validated patient-reported outcome measure grading epiphora severity from 0 (no epiphora) to 4 (epiphora requiring wiping more than 10 times per day) [12]. Primary outcome measures were recorded preoperatively and at each follow-up visit. Due to the variable follow-up periods across cases, data are not presented at uniform timepoints.

For all patients, postoperative complications were assessed as secondary outcomes, including infection, hematoma, wound dehiscence, diplopia, and eyelid malposition recurrence. Skin contour changes along the suture pathway were also assessed at each follow-up visit. Surgically induced astigmatism (SIA) was calculated from cylindrical refractive values obtained preoperatively and at 3 months postoperatively using vector analysis.

Due to the small sample size, descriptive statistics are reported, and no formal statistical analysis was performed.

## 3. Results

### 3.1. Patient Characteristics

Seven consecutive patients (four females, three males; median age 72 years; range 38–86) underwent the procedure during the study period. Patient demographics, diagnoses, anesthesia type, operative time, and concomitant procedures are summarized in Table 1. Indications included post-oncological eyelid reconstruction (*n* = 2; 1 malignant melanoma, 1 sebaceous carcinoma), cicatricial ectropion (*n* = 2), cicatricial entropion (*n* = 1), paralytic ectropion (*n* = 1), and involutional ectropion (*n* = 1). Two patients had a history of prior eyelid surgery. In Case 2, the barbed suture technique was applied to the lower eyelid donor site created by a switch flap used for upper eyelid reconstruction. The median operative time for the barbed suture procedure was 16 min (range 6–23 min); in cases with concomitant procedures (Cases 1, 2, and 3), this reflects the duration of the barbed suture component only.

### 3.2. Clinical Outcomes

**Figure 3:**
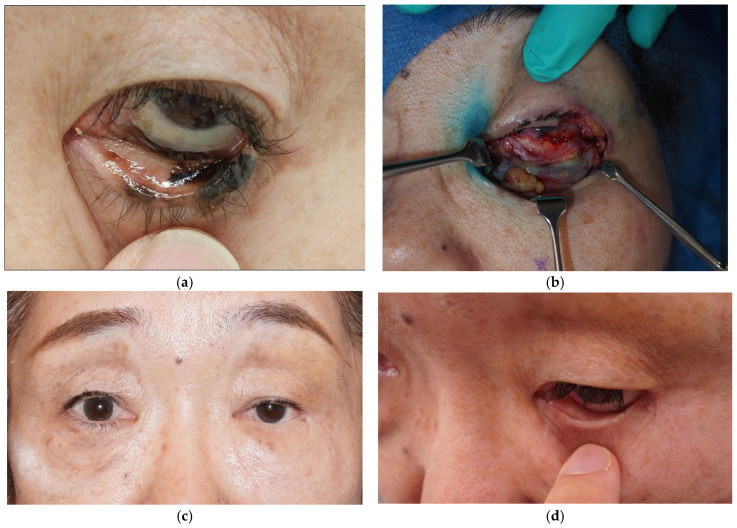
Representative case (Case 1): a 72-year-old woman who underwent total lower eyelid reconstruction following excision of malignant melanoma. (**a**) Preoperative view showing lower eyelid tumor. (**b**) Intraoperative view after tumor excision demonstrating a full-thickness defect. (**c**) Postoperative frontal view at final follow-up (280 days) showing maintained eyelid position. (**d**) Postoperative close-up showing successful graft integration and fornix reconstruction.

The median follow-up period was 189 days (range 105–280). MRD-2 at final follow-up was 5.4 mm in Case 1 (preoperative: 5.7 mm) and 4.1 mm in Case 2 (preoperative: 4.2 mm), indicating maintained eyelid position in both cases. Among the five cases evaluated by the Munk scale, scores improved in four patients. The one patient without improvement (Case 5) had undergone a prior lateral tarsal strip procedure. No recurrence of ectropion or entropion was observed during the follow-up period. Representative clinical photographs of Case 1, in whom total lower eyelid reconstruction was performed using a V-Y advancement cheek flap for the anterior lamella and a combination of lateral orbital periosteal flap and hard palate mucosal graft for the posterior lamella, are presented in Figure 3.

Surgically induced astigmatism was measured in six patients; preoperative measurement was not obtained in one patient (Case 6) due to incomplete data. In Case 7, SIA was 2.01 D (axis 91.5°) at one month postoperatively, which decreased to 0.51 D (axis 180°) at final follow-up, suggesting resolution of transient corneal deformation related to increased eyelid pressure. A summary of outcomes for all patients is provided in Table 2.

### 3.3. Complications

One patient (Case 7) experienced transient postoperative diplopia in the early postoperative period, likely related to increased eyelid pressure on the globe. This resolved with conservative management (lubricating eye drops and observation) within four weeks without further intervention. No cases of infection, hematoma, suture exposure, wound dehiscence, or corneal epithelial disorder were observed.

Transient linear skin indentation along the suture pathway was observed in all seven cases in the early postoperative period. This finding was consistently present at the time of suture removal but resolved spontaneously in all patients by 3 months postoperatively without any intervention. No permanent skin contour irregularity or notching was observed at final follow-up in any case.

## 4. Discussion

This study describes a novel technique for lower eyelid canthal ligament reconstruction using a barbed suture system and reports preliminary clinical outcomes in seven consecutive patients. The principal findings are: eyelid position was maintained in both post-oncological reconstruction cases throughout the follow-up period; Munk scale scores improved in four of five evaluated patients; no recurrence of eyelid malposition was observed; and transient skin indentation along the suture pathway resolved spontaneously in all cases by 3 months.

Lower eyelid malpositions such as ectropion and entropion occur more frequently than in the upper eyelid, partly due to gravitational effects and the relative weakness of the tarsal plate and canthal ligaments [2]. The lower eyelid consists of anterior and posterior lamellae supported by a three-dimensional canthal ligament system that maintains appropriate apposition to the globe. Reconstruction of this support system is therefore essential for successful correction of lower eyelid malposition [1,3].

**Figure 4:**
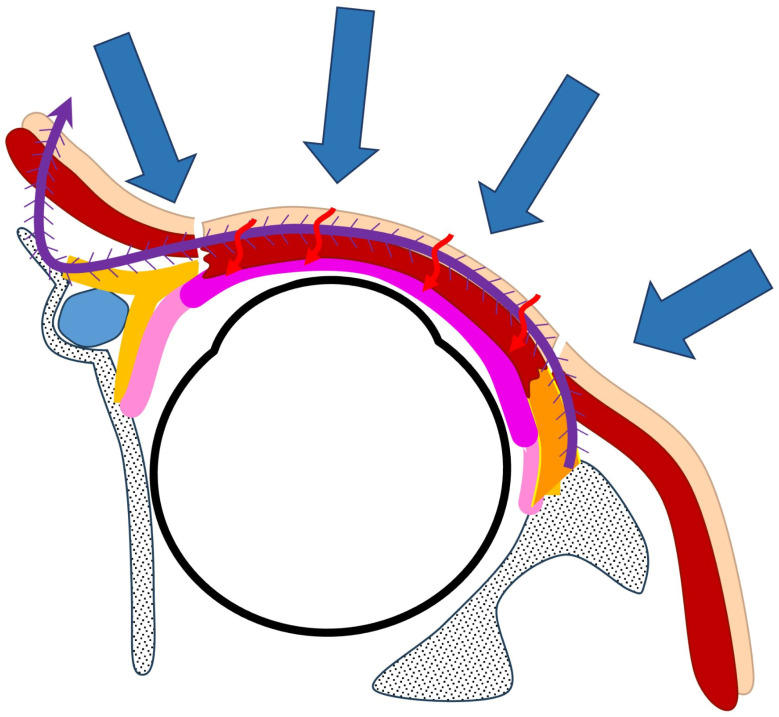
Schematic illustration of the biomechanical and vascular effects of the technique. The barbed suture maintains contact between the anterior lamella and posterior lamellar graft, supporting vascular in-growth (red arrows), while generating tension that promotes eyelid–globe apposition (blue arrow).

In conventional canthal ligament reconstruction, autologous materials such as fascia lata or auricular cartilage are used to reconstruct the lateral and/or medial canthal tendons, providing structural horizontal support [6,7]. However, when canthal ligament reconstruction with a mucosal graft is simultaneously performed, the interposition of avascular graft material between the well-vascularized anterior lamella and the transplanted mucosa may impair vascular ingrowth and reduce graft survival [6]. This remains a hypothesis that has not been directly evaluated in the present study. In contrast, the present technique reconstructs horizontal support using a barbed suture without interposing additional graft material, thereby preserving direct contact between the anterior lamella and the posterior lamellar graft. This may facilitate vascular supply to the mucosal graft and potentially improve graft survival (Figure 4).

A distinctive feature of the present technique is that the barbed suture traverses the entire width of the lower eyelid through defined tissue planes, from the lateral orbital periosteum to the medial canthal periosteum, functioning as a distributed horizontal vector. The barbs are oriented toward the lateral periosteal anchor, such that each engagement point generates a laterally directed traction force while resisting medial displacement—a cumulative distributed lateral suspension effect analogous to that described for barbed sutures in facial lifting procedures [9,13], and representing the key biomechanical advantage over conventional sutures in this application.

Regarding intraoperative tension control, the unidirectional nature of the barbed suture means that tension cannot be released once the suture has engaged tissue. However, the final suture position can be adjusted by varying the depth and extent of the externalization pass at incision ⑤ before the suture is cut. In our experience, this step allows adequate fine-tuning of horizontal support under direct visualization of eyelid margin apposition to the globe. One patient (Case 7) who developed transient diplopia likely had excessive eyelid pressure on the globe, underscoring the importance of careful intraoperative tension assessment.

To our knowledge, barbed sutures have previously been used in the periocular region only as temporary external fixation sutures to prevent ectropion following Mohs micrographic surgery, in which the suture was removed within one week postoperatively [11]. The present technique differs fundamentally in that the barbed suture is used as an absorbable internal reconstruction of the canthal ligament support system, with the suture remaining in situ until absorption.

Eyelid surgery is known to alter corneal biomechanics and may induce refractive changes, including surgically induced astigmatism [14,15]. In the present series, several patients demonstrated transient postoperative changes in astigmatism that stabilized during follow-up, suggesting that eyelid pressure on the corneal surface may temporarily increase following the procedure but ultimately settles within an acceptable range. Careful intraoperative adjustment of suture tension is therefore essential to avoid excessive eyelid pressure.

Because the barbed suture is an absorbable material, concerns may arise regarding the durability of long-term support. Polydioxanone sutures have been reported to induce collagen deposition and tissue remodeling along the implant pathway, and this biological response may persist beyond the period of tensile strength retention [16]. We hypothesize that a similar process along the barbed suture pathway generates secondary fibrous support after suture absorption, effectively replacing mechanical with biological stabilization; however, we emphasize that this remains a biologically plausible hypothesis and should not be interpreted as an inferred mechanism. Although this hypothesis is supported by clinical evidence from barbed PDS sutures used in facial recontouring [16], histological confirmation in the periocular setting is lacking, and prospective validation is required. In the present series, four cases (Cases 1, 2, 3, and 7; range 189–280 days) were observed beyond the expected period of complete suture absorption with no recurrence, providing preliminary support for this concept. Nevertheless, when used alone for cicatricial, paralytic, or severe degenerative malpositions, insufficient long-term support and late recurrence remain potential concerns, and careful patient selection is warranted.

The indications and limitations of this technique warrant careful consideration. The present technique is best suited for cases in which the posterior lamella has been reliably reconstructed—for example, post-oncological reconstruction cases with palatal mucosal grafting—as well as in cases of mild-to-moderate ectropion of various etiologies. Because the technique provides linear rather than sheet-like structural support, it may be insufficient as a standalone procedure in cases of severe eyelid laxity with near-complete tarsal disruption, or paralytic ectropion with significant orbicularis weakness. In the present series, the patient with paralytic ectropion (Case 6) showed only partial improvement (Munk scale 2 → 1), and the patient with prior lateral tarsal strip surgery (Case 5) showed no additional improvement. Compared with conventional approaches such as the lateral tarsal strip procedure, the present technique does not require tarsal manipulation or tissue shortening and provides support across a wider horizontal extent of the eyelid; however, it does not provide the rigid structural reinforcement afforded by autologous grafts such as fascia lata or auricular cartilage. The present technique is therefore best viewed as complementary to, rather than a replacement for, established methods, and may be particularly suited to cases in which donor-site morbidity is to be avoided or in which canthal ligament reinforcement is required as an adjunct to anterior and posterior lamellar reconstruction.

Transient linear skin indentation along the suture pathway was observed in all seven cases in the early postoperative period, consistently present at the time of suture removal. This finding is attributable to the mechanical tethering effect of the barbed suture on the overlying subcutaneous tissue and skin and resolved spontaneously in all patients by 3 months postoperatively. Because this change resolved uniformly and predictably, we consider it an expected feature of the postoperative course rather than a complication. Surgeons employing this technique should counsel patients regarding this transient appearance in advance.

This study has several limitations. First, the sample size was small, comprising only seven cases, which limits the generalizability of the findings. Second, three cases (Cases 4, 5, and 6) had follow-up periods of 105–122 days. According to the manufacturer’s Instructions for Use, STRATAFIX^®^ Spiral PDS Plus^®^ (4-0) retains approximately 50% of its original tensile strength at 4 weeks and 37% at 6 weeks, with absorption essentially complete at 210 days post implantation (data on file). Given the progressive decline in tensile strength beyond 6 weeks, it is reasonable to infer that by 105 days (15 weeks)—the minimum follow-up in this series—residual tensile strength would be negligible. On this basis, the observed eyelid stability in all cases is more consistent with biological tissue consolidation along the suture pathway than with ongoing mechanical suture support. Nevertheless, we acknowledge that the absence of histological confirmation and the relatively short follow-up represent genuine limitations, and prospective studies with follow-up beyond 12 months are required to definitively establish long-term durability. Third, this was a single-center, single-surgeon retrospective study without a comparative control group (e.g., lateral tarsal strip or graft-based techniques), which precludes comparative efficacy assessment and introduces potential selection bias. Fourth, the patient cohort was heterogeneous, comprising five distinct indications with fundamentally different pathophysiology; outcomes across these subgroups are not directly comparable. Fifth, only descriptive statistics were reported due to the small sample size. Further prospective studies with larger cohorts, longer follow-up, and comparative designs are required to validate the efficacy and durability of this technique.

## 5. Conclusions

This preliminary case series demonstrates the feasibility of lower eyelid canthal ligament reconstruction using a barbed suture system, with encouraging short-term outcomes and no recurrence of eyelid malposition during the follow-up period. The most promising indications appear to be post-oncological reconstruction and mild-to-moderate ectropion support; applicability across all etiologies remains to be established. Larger, prospective, controlled studies with longer follow-up are required to establish long-term efficacy and durability.

## Figures and Tables

**Table 1:** Patient characteristics.

Case	Age (Years)	Sex	Side	Anesthesia	Op. Time (min) §	Diagnosis	Etiology	Concomitant Procedures	Previous Eyelid Surgery
1	72	F	L	GA	9	Total lower eyelid defect (malignant melanoma) †	Tumor resection	V-Y advancement flap; lateral orbital periosteal flap; hard palate mucosal graft	None
2	77	F	R	LA	6	Lateral lower eyelid defect (sebaceous carcinoma) ‡	Tumor resection	Switch flap; V-Y advancement flap; lateral orbital periosteal flap; hard palate mucosal graft	None
3	38	M	L	GA	16	Lower eyelid entropion	Cicatricial	Tarsal repair	None
4	50	M	L	LA	23	Lower eyelid ectropion	Cicatricial	None	Skin graft
5	86	F	R	LA	15	Lower eyelid ectropion	Cicatricial	None	Lateral tarsal strip
6	41	F	R	LA	18	Lower eyelid ectropion	Paralytic	None	None
7	77	M	L	GA	20	Lower eyelid ectropion	Involutional	None	None

† Primary tumor of the lower eyelid. ‡ Primary tumor of the upper eyelid; the barbed suture technique was applied to the lateral lower eyelid donor site created by a switch flap. § Operative time definition varies by case; see Section 2.2. R: right; L: left; F: female; M: male; GA: general anesthesia; LA: local anesthesia.

**Table 2:** Clinical outcomes.

Case	MRD-2 Pre → Post (mm)	Munk Scale Pre → Post	SIA (D)	SIA Axis (°)	Complications	Follow-Up (Days)
1	5.7 → 5.4	–	1.39	54.2	None	280
2	4.2 → 4.1	–	0.25	11	None	196
3	–	4 → 0	0.51	165.9	None	189
4	–	3 → 2	0.25	88	None	122
5	–	3 → 3	0.72	139.9	None	119
6	–	2 → 1	N/A †	N/A †	None	105
7	–	2 → 0	0.51 ‡	180.0 ‡	Transient diplopia	238

† Preoperative measurement not obtained. ‡ Value at final follow-up; SIA at 1 month postoperatively was 2.01 D (axis 91.5°). MRD-2: margin reflex distance 2; SIA: surgically induced astigmatism; –: not applicable.

## Data Availability

Data are available upon reasonable request to the corresponding author.

## Abbreviations

The following abbreviations are used in this manuscript: MRD-2: Margin reflex distance 2; SIA: Surgically induced astigmatism; PDS: Polydioxanone suture; ICC: Intraclass correlation coefficient.
